# Host mediated inflammatory influence on glioblastoma multiforme recurrence following high-dose ionizing radiation

**DOI:** 10.1371/journal.pone.0178155

**Published:** 2017-05-22

**Authors:** J. Tyson McDonald, Xuefeng Gao, Cole Steber, Jawon Lee Breed, Caitlin Pollock, Lili Ma, Lynn Hlatky

**Affiliations:** 1 Center of Cancer Systems Biology, Boston, Massachusetts, United States of America; 2 Cancer Research Center, Hampton University, Hampton, Virginia, United States of America; 3 Shenzhen HRK Bio-tech Co., Ltd, Shenzhen, Guangdong, China; Northwestern University Feinberg School of Medicine, UNITED STATES

## Abstract

Despite optimal clinical treatment, glioblastoma multiforme (GBM) inevitably recurs. Standard treatment of GBM, exposes patients to radiation which kills tumor cells, but also modulates the molecular fingerprint of any surviving tumor cells and the cross-talk between those cells and the host. Considerable investigation of short-term (hours to days) post-irradiation tumor cell response has been undertaken, yet long-term responses (weeks to months) which are potentially even more informative of recurrence, have been largely overlooked. To better understand the potential of these processes to reshape tumor regrowth, molecular studies in conjunction with *in silico* modeling were used to examine short- and long-term growth dynamics. Despite survival of 2.55% and 0.009% following 8 or 16Gy, GBM cell populations *in vitro* showed a robust escape from cellular extinction and a return to pre-irradiated growth rates with no changes in long-term population doublings. In contrast, these same irradiated GBM cell populations injected *in vivo* elicited tumors which displayed significantly suppressed growth rates compared to their pre-irradiated counterparts. Transcriptome analysis days to weeks after irradiation revealed, 281 differentially expressed genes with a robust increase for cytokines, histones and C-C or C-X-C motif chemokines in irradiated cells. Strikingly, this same inflammatory signature *in vivo* for *IL1A*, *CXCL1*, *IL6* and *IL8* was increased in xenografts months after irradiation. Computational modeling of tumor cell dynamics indicated a host-mediated negative pressure on the surviving cells was a source of inhibition consistent with the findings resulting in suppressed tumor growth. Thus, tumor cells surviving irradiation may shift the landscape of population doubling through inflammatory mediators interacting with the host in a way that impacts tumor recurrence and affects the efficacy of subsequent therapies. Clues to more effective therapies may lie in the development and use of pre-clinical models of post-treatment response to target the source of inflammatory mediators that significantly alter cellular dynamics and molecular pathways in the early stages of tumor recurrence.

## Introduction

The standard of care for newly diagnosed glioblastoma multiforme (GBM) is a multi-modality strategy beginning with maximal surgical resection followed by fractionated radiotherapy, (60Gy, 30–33 fractions of 1.8–2.0Gy) with temozolomide given during and after the irradiation [1]. In clinical trials, this strategy increased the median survival of GBM patients from 12.1 to 14.6 months, as well as increased the two-year survival rate from 10.4% to 26.5%. Though widespread adoption of this multimodality therapy has had meaningful benefit in the clinic, tumor recurrence remains the major challenge, especially in individuals age 80 and older [2, 3]. To treat recurrent brain tumors, stereotactic radiosurgery is an option used for previously irradiated primary brain tumors as well as brain metastases. This therapy uses high, single fraction doses of ionizing radiation (IR) to target the tumor volume. The maximum tolerated single fraction dose for radiosurgery is recommended as 15Gy for tumors 31-40mm, 18Gy for tumors 21-30mm, and 24Gy for tumors less than 20mm in diameter [4].

The majority of patients diagnosed with GBM will undergo radiation therapy as part of their clinical treatment. Despite an initial positive response, the nearly inevitable recurrence of the tumor is often attributed to therapeutic resistance including radiation-resistance, given that most recurrent GBMs are found within the prescribed radiation treatment volume, and the failure of radiation dose escalation to improve responses [5, 6]. Following irradiation, surviving cells and their progeny are often characterized as having a delayed non-clonal appearance with chromosomal aberrations, micronuclei, genetic mutations and enhanced cell death, generally referred to as radiation-induced genomic instability (reviewed by [7]). Current radiobiological understanding of the molecular and cellular response to IR is largely focused on the short-term period of hours to days following radiation exposure, as such these pre-clinical studies have contributed significantly to the success of radiation therapy in exerting considerable short-term tumor control. To address the long-term radiation response of GBM, we investigated how irradiation modulates the cellular dynamics and molecular signaling within GBM cell populations from days to months post-irradiation. Our findings provide insight into the evolution of GBM cell populations under the selective pressure of irradiation, suggesting critical determinates for successfully achieving cellular extinction within the primary and the recurrent tumor sub-populations. The experiments presented here examined long-term *in vitro* cellular responses, proliferation, and genome-wide transcriptome expression, as contrasted with *in vivo* xenograft growth dynamics and gene expression signatures of tumors grown from these irradiated GBM cell populations, to additionally gain insight into the role of the host in modifying tumor radio-response. Quantitative analysis via a cellular automaton model constructed using this data as a framework, points to the intervention and manipulation of the tumor cell-host environment days to weeks following the radiation insult as critical to slowing tumor growth, to quell a resurgence in post-irradiation tumor-population doubling.

## Materials and methods

### Ethics statement

Animal tumor model studies were performed in strict accordance with the recommendations in the Guide for the Care and Use of Laboratory Animals of the National Institutes of Health. Protocols used were approved by the Institutional Animal Care and Use Committee (IACUC) at Tufts University School of Medicine for studies using human U87-MG cells (Protocol: #P11-324). Institutions are AAALAC accredited and every effort was made to minimize animal distress.

### *In vitro c*ulture and irradiation

Previous studies from our team’s *in silico* modeling parameterized by biological measurements has led to increased understanding of the radiation response in glioma stem cell division and of cellular reprogramming following irradiation [8–10]. Using this integrated computational/wet-lab approach, we have added additional parameterization to explore tumor recurrence using the human U87-MG glioblastoma cell line (ATCC, Bethesda, MD). The human U87-MG glioblastoma cell line (ATCC, Bethesda, MD) was cultured in MEM (Life Technologies, Grand Island, NY) with 10% Fetal Bovine Serum (Lonza, Hopkinton, MA), maintained in a humidified 37°C incubator with 5% CO_2_, and authenticated by short-tandem repeat profiling. Cultures were treated with single doses of IR between 0 and 16Gy (Cesium-137 Gammacell irradiator) with a dose-rate of 0.48Gy per minute. For short-term radiobiological endpoints, cells were plated overnight before treatment and harvested at days 1, 4 or 6 post-irradiation. For the long-term endpoints, post-irradiation cells were harvested every 4 to 5 days for counting, viability testing and re-plating. A total of three independent experimental replicates were used at days 34, 35 and 36 post-irradiation and are referred collectively as “day 35”. Trypan-blue negative (viable) cells were measured using a Cellometer Auto T4 Cell Counter (Nexcelom Bioscience, Lawrence, MA).

### Post-irradiation *in vivo t*umor formation

U87-MG cells were plated overnight following exposure with acute doses of 8Gy (N = 8), 12Gy (N = 7) or 16Gy (N = 10), or by sham irradiation, 0Gy (N = 7). At 24 hours after the irradiation 2×10^6^ viable cells were subcutaneously injected into the caudal portion of the back of eight-week old male nude mice (Foxn1^nu^, Jackson Labs, Bar Harbor, ME). Animal weight and tumor size was measured daily. Mice were sacrificed when tumors reached 1500cm^2^. At necropsy tumor samples were snap frozen for RNA extraction with TRIzol (Life Technologies) and whole cell lysates made for western blotting.

### Post-irradiation population survival and cellular division

A standard of the radiation field, the clonogenic assay, was used to assess cell survival as a function of radiation dose as previously described [11]. Cell division following irradiation was also measured in the clonogenic assay using the lipophilic dye PKH-26 as instructed by the manufacturer (Sigma-Aldrich, St. Louis, MO). Cells were stained with 30μM and plated overnight before irradiation. Following colony formation, single cell suspensions were analyzed by flow cytometery on the FC500 (Beckman-Coulter, Brea, CA). The number of cell divisions was analyzed using ModFit LT3.2 software.

### Transcriptome expression

Genome-wide gene expression profiling was performed using HumanHT-12v4 expression bead chips on the Illumina iScan (Illumina, San Diego, CA) as previously described [11]. At days 1, 4, 6 or 35 after glioblastoma cell population exposures of 0, 8 or 16Gy, total RNA was collected using TRIzol (Life Technologies). A total of 3 independent replicates were used for short-term samples on days 1, 4, and 6 resulting in 27 microarrays (3 conditions × 3 replicates × 3 time points) while samples on day 35 used four experimental replicates resulting in an additional 12 microarrays for a total of 39 microarrays.

### Expression data pre-processing

The signal intensities for 47,300 total probes were exported with the GenomeStudio’s Gene Expression module version 1.6.0 (Illumina). Quantile normalization and log_2_ transformation was performed separately for short- (day 1, 4 and 6) and long- (day 35) term irradiations using GenePattern [12]. Short-term samples were also processed with ComBat to remove non-biological batch effects [13]. The dataset was collapsed to 31,322 unique genes and differential expression was measured using a one-way ANOVA followed by correction for multiple hypothesis testing. Using 12 groups by irradiation and time point resulted in 3,213 genes with a Bonferroni-Holm false discovery rate (FDR) less than 10% of which 281 genes displayed greater than ±1.5 fold-change compared to pre-irradiated time points (S1 Table). Microarray data is available at the Gene Expression Omnibus (GEO) repository under accession number GSE56937.

### Microarray data analysis

The 281 statistically significant genes were uploaded to DAVID version 6.7 resulting in a 273 gene list with 8 unannotated genes [14, 15]. Functional annotation clustering was used to measure the relationships between annotated terms and the degree of similarity to the input gene list. The calculated EASE p-value is a modified Fisher Exact p-value to determine the gene-enrichment within the annotation terms. Redundant annotations are clustered together based on similar gene members using high stringency resulting in an overall rank enrichment of the annotation term group based on the geometric mean of the cluster EASE scores. Functional gene classification was used to cluster gene-to-gene similarity with shared functional annotations.

Characterization of the pre-processed list of 31,322 genes for each experimental condition relative to the pre-irradiated control was performed using Gene Set Enrichment Analysis (GSEA) [16]. Samples were compared to the Gene Ontology C5 collection in the Molecular Signatures Database containing more than 1400 gene sets. Results indicated a significant number of gene sets with a statistically conservative familywise error rate (FWER) correction less than 5% (S2 Table). The Enrichment Map plug-in for cytoscape was used to visualize related gene sets [17].

### Quantitative RT-PCR and western blotting

Validation of gene expression was performed on the 7500 Real-Time PCR System (Applied Biosystems, Carlsbad, CA). After normalization to a multiplexed 18S housekeeping probe, gene expression for the target probe was calculated using the 2^-ΔΔCt^ method relative to the pre-irradiated control. All probes were purchased from Applied Biosystems. Protein expression via western blotting was performed as previously described [18] using 10μg of total protein extracted from tissue lysates from *in vivo* tumor experiments at 0, 8 and 12Gy using IL6 and IL8 antibodies (Abcam, Cambridge, MA).

### *In silico* cellular automaton model of post-irradiation tumor dynamics

A cellular automaton model based on rules for independent cells in response to their local environment was used to model tumor growth as previously published [8, 9]. Three cell states were defined: proliferative, quiescent (or transiently arrested) and non-proliferative (stably arrested) but metabolically active (NPMA). The addition of the NPMA phenotype from our previous model fits with the previously observed senescence-associated secretory phenotype (SASP) [19]. The SASP phenotype has been implicated as both tumor suppressive and promoting, is associated with inflammation and may be acquired following high dose IR exposure [20, 21]. Previously estimated model parameterization for migration frequency (*μ)*, cellular maturity (*m*), probability of cell death per day (*p*_*a*_) and probability to undergo mitosis (*p*_*d*_) were used as described by Gao, *et al*. [8]. The extension of this model to the case following radiation exposure adds a NPMA state due to some irradiated cells losing the ability to divide but remaining viable. These cells are distinguished from other forms of radiation-induced inactivation (*i*.*e*. cell death) with a probability of becoming a NPMA cell (*p*_*s*_) versus the probability of death (1-*p*_*s*_). Continuous tumor growth is allowed in the context of the bulk environmental carrying capacity constraints in aggregate (*e*.*g*. extracellular matrix, vasculature and immunity).

## Results

### Following high-dose radiation exposure, long-term GBM cell population growth dynamics show an escape from extinction and restoration of the dynamics existing in the pre-irradiated state

**Fig 1:**
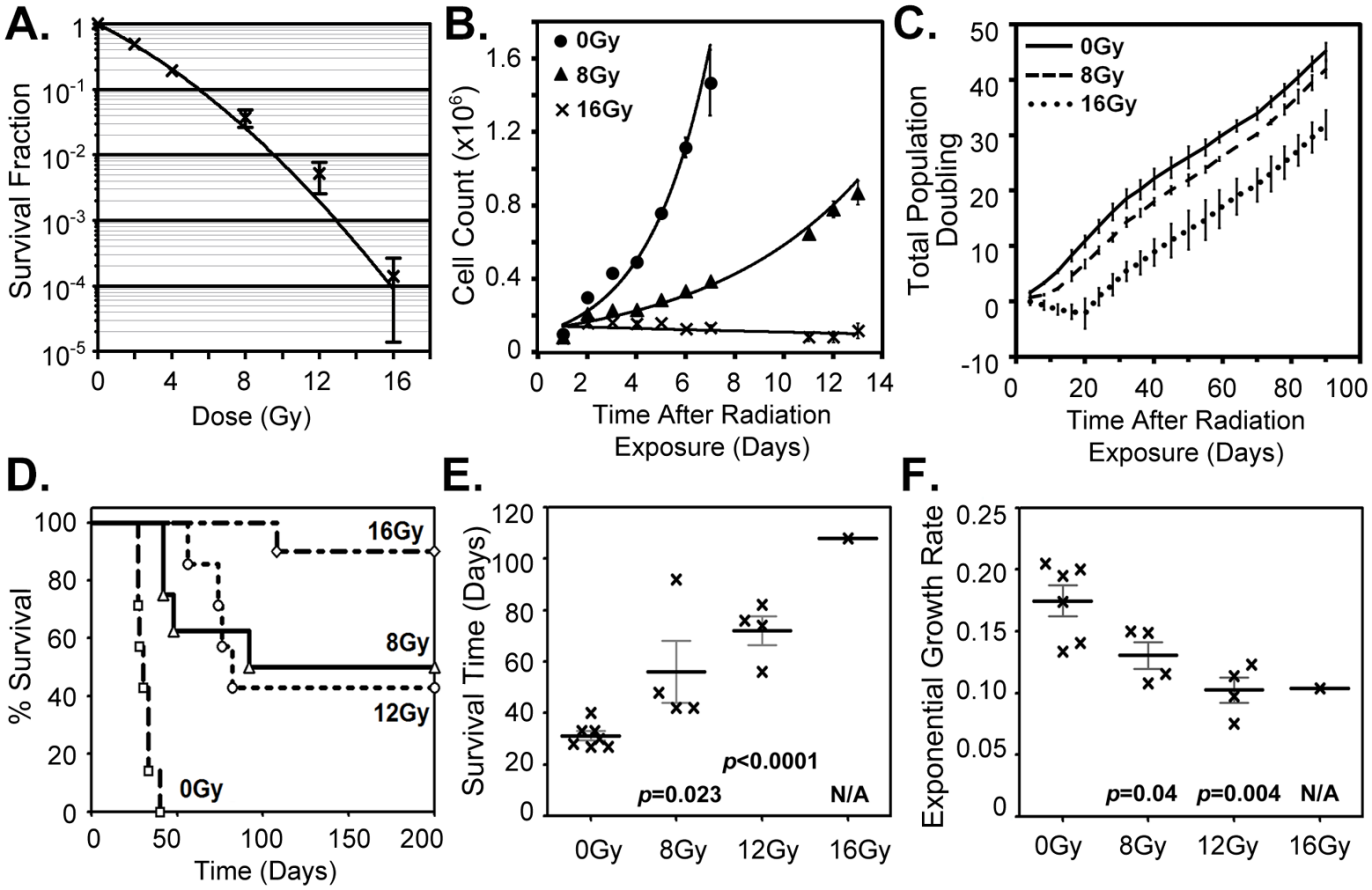
Short- and long-term GBM cell population response to ionizing radiation. (A.) The fraction of U87-MG cells with clonogenic capacity following 0 to 16Gy. N = 4, SEM is shown. (B.) Irradiated cell population short-term growth dynamics demonstrates a reduced ability to sustain the GBM cell population. Cells plated immediately after exposure to 0, 8 or 16Gy. N = 2, SD is shown. (C.) Examination of the long-term population doubling dynamics following 0, 8 or 16Gy. The cell population doubling rates for both 8 and 16Gy populations were observed to rebound to essentially that of the original pre-irradiated GBM cell population. N = 3, SD is shown. (D. to E.) Data on U87-MG xenografts grown from subcutaneous injection of 2x10^6^ U87-MG cells 24 hours after *ex vivo* irradiation of 0, 8, 12 or 16Gy. (D.) Kaplin-Meier survival of mice injected with control 0Gy (0 of 7) or irradiated U87-MG cells receiving 8Gy (4 of 8), 12Gy (3 of 7) or 16Gy (9 of 10). (E.) Average survival time for 0Gy, 8Gy, 12Gy and 16Gy. (F.) *In vivo* growth rates of “recurrent” tumors, grown from post-irradiated cells, to exponential fits above 200mm^3^. *p-values* are compared to 0Gy controls.

Acute delivery of high dose IR to GBM cell populations were used to investigate how the dynamics of those surviving GBM cells are perturbed post-irradiation. In doing so, we consider that it is the surviving cells that escape population extinction and give rise to any tumor recurrence. To study the rebounding population after high doses of IR, measures of clonogenic survival were used to determine the dose necessary to achieve a minimal survival probability that, never-the-less, maintained long-term reproductive capacity for the U87-MG cell populations. Fitting to the established linear-quadratic model resulted in a population survival of 2.55% at 8Gy and 0.009% at 16Gy (Fig 1A). Resolution of the structure of the short-term growth dynamics following 8 or 16Gy, was examined *in vitro* by monitoring cell population growth over two weeks (Fig 1B). A marked reduction in the growth capacity of the irradiated cell populations was detected, though an exponential growth rate was still observed for the 8Gy irradiated samples (R^2^ = 0.9182) compared to the 0Gy control (R^2^ = 0.9299). However, no growth capacity was evident for the irradiated population with 16Gy (R^2^ = 0.1651) and the number of viable cells was relatively constant over this same two-week period. These findings are consistent with, and these investigations augment, considerable previous *in vitro* studies on GBM cellular response to IR that typically monitor cells (*e*.*g*. expression of cell cycle checkpoint genes for execution of DNA repair) for short periods of time after exposure.

To gain critical insight on the inevitable regrowth of GBM cell populations following radiation treatment, we shifted focus to the examination of long-term response by measuring the proliferative capacity and molecular modulation of the surviving irradiated cells and their progeny over extended post-irradiation periods. To this end, GBM cells, irradiated with 0, 8 or 16Gy, were cultured and counted every 4 to 5 days for up to 90 days (Fig 1C). At 4 and 8 days after irradiation, decreased population growth was again clearly evident in the 8Gy and 16Gy samples. Yet, strikingly, after approximately one week the 8Gy samples recovered their original proliferative capacity and exhibited the population doubling time of the pre-irradiated population. Similarly, although the proliferative capacity of the 16Gy irradiated samples (with 0.009% survival), decreased for up to three weeks post-irradiation (days 4 to 20), the population self-restored its apparent full proliferative capacity and also achieved a growth rate comparable to that of the pre-irradiated GBM cell population. These irradiated populations maintained this restored pre-irradiated growth rate for the duration of the time measured, three months. Notably, these results suggest that the large cytotoxic selection pressures, exerted on GBM cell populations due to exposure to high doses of IR, fail to significantly alter the intrinsic growth dynamics of the recurrent GBM cell population over the long run (as measured *in vitro*). Such findings highlight the need to fully understand such tumor population-level restorative mechanisms that can compensate for, and thereby rival, the cell-level killing action of irradiation.

To contrast these findings with *in vivo* growth dynamics of the viable progeny from the irradiated GBM cell population, a tumor xenograft model was established. U87-MG cell populations were irradiated *ex vivo* with 0, 8, 12 or 16Gy at 24 hours prior to the implantation of the equivalent numbers of viable cells for each condition. This model allows direct time-dependent measurement of the tumor volume, as well as isolation of the radiation-response of the tumor cell population under investigation, without the confounding damage to the surrounding normal tissue as is unavoidable when irradiating tumors *in situ* (e.g. tumor bed effect). This permitted our observation of changes emanating directly from the irradiated GBM cell population, along with facilitating the integration *in vivo* responses with an *ex vivo* radiation, which complements the parallel *in vitro* studies. Tumor formation was observed in 7 of 7 mice for GBM cells irradiated with 0Gy, 4 of 8 mice for cells irradiated with 8Gy, 4 of 7 mice for cells irradiated with 12Gy and 1 of 10 mice for cells irradiated with 16Gy (Fig 1D). In contrast to the restoration of population growth rates demonstrated for the these same cells *in vitro*, following an initial period of growth inhibition post-irradiation, fitting the individual *in vivo* exponential growth rates above 200mm^3^ tumor volumes demonstrated a statistically significant decrease in the growth potential of the irradiated population occurred with dose (Fig 1F). The noteworthy discrepancy, between the *in vitro* and *in vivo* population growth rate data post-irradiation, demonstrates the *in vivo* host system critically modulates the intrinsic proliferative potential of the surviving GBM cells, resulting in this case in a significantly reduced capacity for *in vivo* regrowth as compared to that of the control 0Gy tumor population.

### *In vitro* gene expression of high-dose-irradiated GBM cells reveals a robust inflammatory response prior to population recovery with echoes of a radiation damage response after escape from cellular extinction

**Fig 2:**
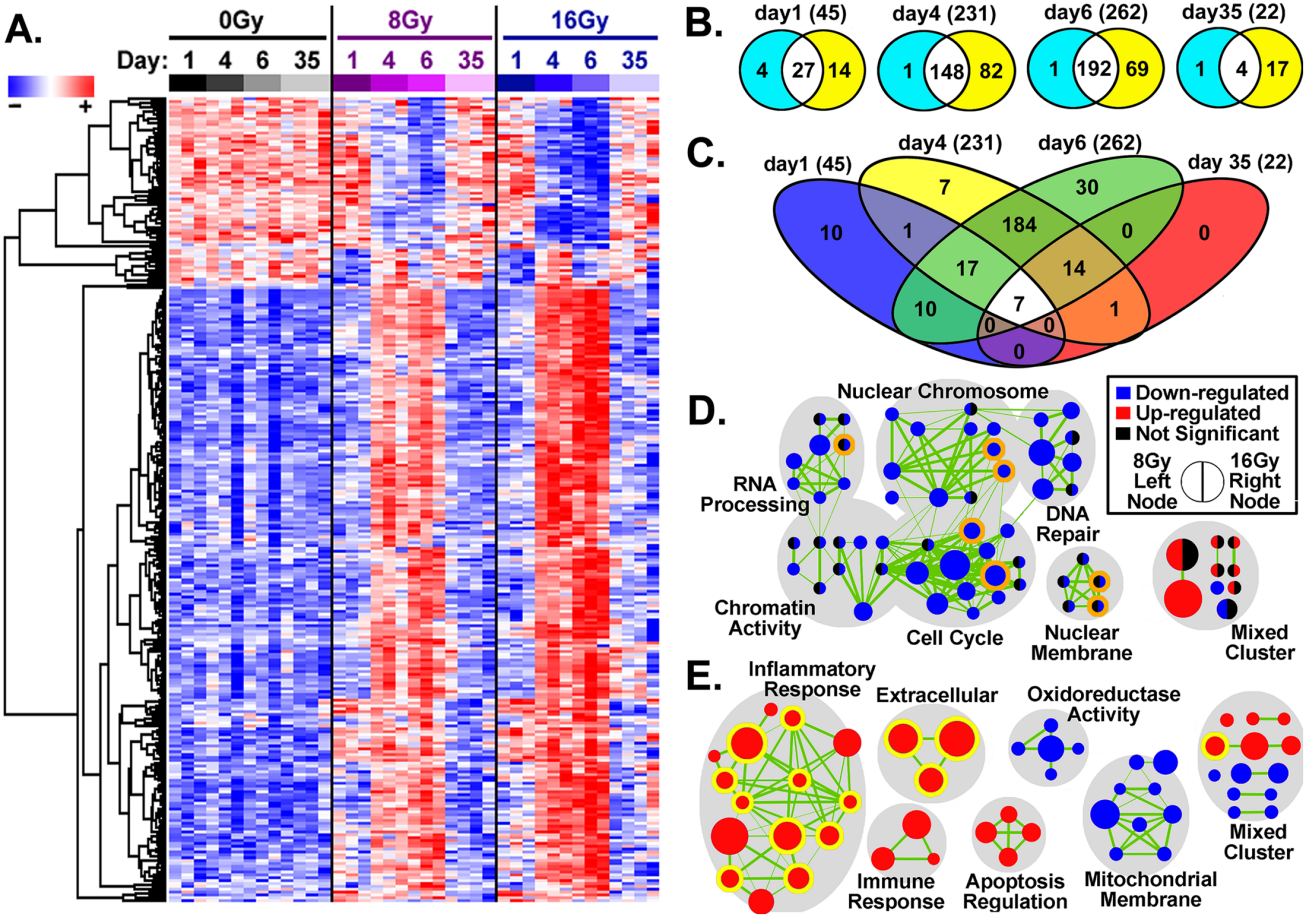
Genome-wide expression analysis of the U87-MG cell population at 1, 4, 6 and 35 days after irradiation. (A.) Time and dose dependence of hierarchical gene clustering using an Euclidean algorithm for the 281 genes with statistically significant differential regulation. Note the considerable overall dysregulation pattern detected for these genes at days 4 and 6 (to a lesser extent at day 1) post-irradiation, is seen to largely self-correct by day 35. (B.) Number of statistically significant genes by day comparing 8Gy (turquoise/white fills) versus 16Gy (yellow/white fills). Note 16Gy consistently regulated more genes at each time point. (C.) Number of statistically significant genes by day with unique genes after combining results from 8Gy and 16Gy gene lists. Number of unique genes are shown in parentheses. (D. & E.) GSEA overlapping gene sets as a network connectivity maps for similar gene sets (nodes) were created for (D.) day 1 and (E.) day 4. A robust inflammatory signature is observed days following irradiation corresponding to the decreased population doubling of the irradiated cell populations. Following escape from extinction (day 35), a lesser response in common with the early exposure (day1) is observed. Node size is indicative of the number of genes in the set, while edge thickness (green lines) is proportional to the gene overlap between gene-sets. There were 7 genes sets in common between days 1 and 35 (orange outlined nodes) and 14-gene sets in common between days 4 and 6 (yellow outlined nodes).

The short-term molecular response to high-dose IR exposure was explored in GBM cell populations *in vitro*, with genome-wide expression microarrays run at 1, 4 or 6 days after irradiation with 0, 8 or 16Gy. In addition, expression data at 35 days after IR exposure was also collected and examined at which a resurgence of cell population doubling indicated a recovery growth rate similar to that of the pre-irradiated control population. Following high-dose exposure, a total of 281 genes were found with a significant differential expression compared to pre-irradiated control samples (FDR <10% and >±1.5-fold change; Fig 2A and S1 Table). Regardless of the details of the dose (8 vs 16Gy), the irradiated GBM cell populations exhibited a strong time-dependent perturbation in gene expression peaking 6 days after exposure (Fig 2B and 2C). At day 1, the earliest time examined, expression of well-known radiation-related genes were observed such as cyclin-dependent kinase inhibitor 1A (*CDKN1A*), DNA-damage-inducible transcript 4-like (*DDIT4L*) and critical cell cycle regulator cyclin A2 (*CCNA2*). Yet, the greatest alteration of overall transcription was observed on days 4 and 6 following radiation exposure with considerable gene overlap observed on these days (184 of 281 significantly expressed genes). At longer times post-exposure, day 35, considerably fewer genes were determined to be significantly expressed, with 5 and 21 genes for 8 and 16Gy respectively. Noteworthy were a unique set of 7 genes found to be significantly dysregulated at every time point investigated: *C1QTNF1*, *C9ORF169*, *IGFBP1*, *IL1B*, *IL8*, *PI3*, and *RRAD* (Fig 2C).

To understand the functional relevance of the list of aggregated genes, GSEA and network mapping were used as an independent method to interpret the biological significance, find coordinated alteration in functional pathways and provide increased temporal understanding [16]. The resulting network on day 4 was highly similar to that of day 6 while day 35 contained too few connective gene sets to make a functional network. Thus, the clustering of similar biological networks were created for days 1 and 4 post-irradiation and overlapping gene sets for days 6 and 35 were highlighted (Fig 2D and 2E). Examination of the most common gene sets in these networks reveals a temporal pattern shifting away from regulation of the classic radiation-induced genetic response of DNA damage repair and cell cycle arrest (Fig 2D), to exhibit a strong up-regulated inflammatory response accompanied by down-regulation in cellular mitochondrial membrane functions at day 4 (Fig 2D). From a temporal perspective, of the 14 up-regulated gene sets found on days 4 and 6, regardless of dose, 10 of these gene sets were involved in inflammatory responses (Fig 2E, yellow outlined nodes). Interestingly, at day 35 in the long-term cell cultures that escaped growth extinction, there remained 7 down-regulated gene sets in common with those of day 1, suggesting a long-lasting perturbation in the surviving progeny from radiation exposure.

To better pinpoint the critical regulators of the inflammatory response, the DAVID database was used to reveal over-represented gene families and clusters according to biological function. Three major gene families were involved, the interleukin-1 (*IL1*), histone and chemokine families (S3 Table). Functional annotation clustering identifying statistically significant overlapping pathways found dysregulation of *IL1*, chromatin/nucleosome assembly and chemokine family members as well as regulation of genes involved in cell death (Table 1). The robust increase in *IL1* related genes observed, strongly suggests a linked coordinated expressions of a radiation-induced inflammatory module, as 7 out of the 9 *IL1* family genes significantly expressed cluster together on a 400kb region of chromosome 2 [22].

**Table 1:** Functional annotation clustering using DAVID of the identifiable 273 statistically significant genes with a Bonferroni FDR correction for multiple hypothesis testing less than 5%.

Terms	Category^a^	Enrichment Score^b^	Genes
IL-1, IL-1 receptor binding	IPR000975	8.108	*IL1A*, *IL1B*, *IL1F5 (IL136RN)*, *IL1F8 (IL36B)*, *IL1F9 (IL36G)*, *IL1F10*, *IL1F3 (IL1RN)*
	SM00125		
	GO:0005149		
Nucleosome assembly and organization, protein-DNA complex assembly, chromatin assembly or disassembly	GO:0006334	6.631	*H1F0*, *H2AFJ*, *HIST2H2AA3*, *HIST1H2AC*, *HIST2H2AC*, *HIST1H2BD*, *HIST1H2BG*, *HIST1H4C*, *HIST2H2BE*, *HJURP*, *HMGB2*, *TSPYL2*
	GO:0031497		
	GO:0065004		
	GO:0034728		
	GO:0006333		
Small chemokine, IL-8-like, SCY, chemokine activity, chemokine receptor binding, chemokine signaling pathway	IPR001811	5.528	*CCL2*, *CCL3*, *CCL3L1*, *CCL3L3*, *CCL5*, *CCL20*, *CXCL1*, *CXCL2*, *CXCL5*, *IL8*
	SM00199		
	GO:0008009		
	GO:0042379		
Regulation of apoptosis, regulation of programmed cell death, regulation of cell death	GO:0042981	4.763	*ADORA2A*, *ANGPTL4*, *BCL11B*, *CARD10*, *CCL2*, *CDKN1A*, *CITED2*, *CLCF1*, *CSF2*, *DDIT3*, *DEDD2*, *DUSP1*, *ERN1*, *FOSL1*, *IER3*, *IL12A*, *IL1A*, *IL1B*, *IL6*, *NQO1*, *NRG1*, *PTGS2*, *SCG2*, *SERPINB2*, *SOD2*, *SPHK1*, *TGM2*, *TNFAIP3*, *TNFRSF10B*, *TOP2A*, *TXNIP*
	GO:0043067		
	GO:0010941		
Negative regulation of apoptosis, programmed cell death and cell death	GO:0043066	4.687	*ADORA2A*, *ANGPTL4*, *BCL11B*, *CCL2*, *CDKN1A*, *CITED2*, *CLCF1*, *CSF2*, *IER3*, *IL1A*, *IL1B*, *IL6*, *NRG1*, *SCG2*, *SERPINB2*, *SOD2*, *SPHK1*, *TGM2*, *TNFAIP3*
	GO:0043069		
	GO:0060548		

^a^INTERPRO (IPR), SMART (SM) or Gene Ontology (GO) Pathways.

^b^Geometric mean of group significance with pathway EASE scores (modified Fisher exact p-value); -log scale.

### Sustained inflammatory cytokine and chemokine expression in post-irradiation “recurrent” tumor xenografts long after radiation exposure

**Fig 3:**
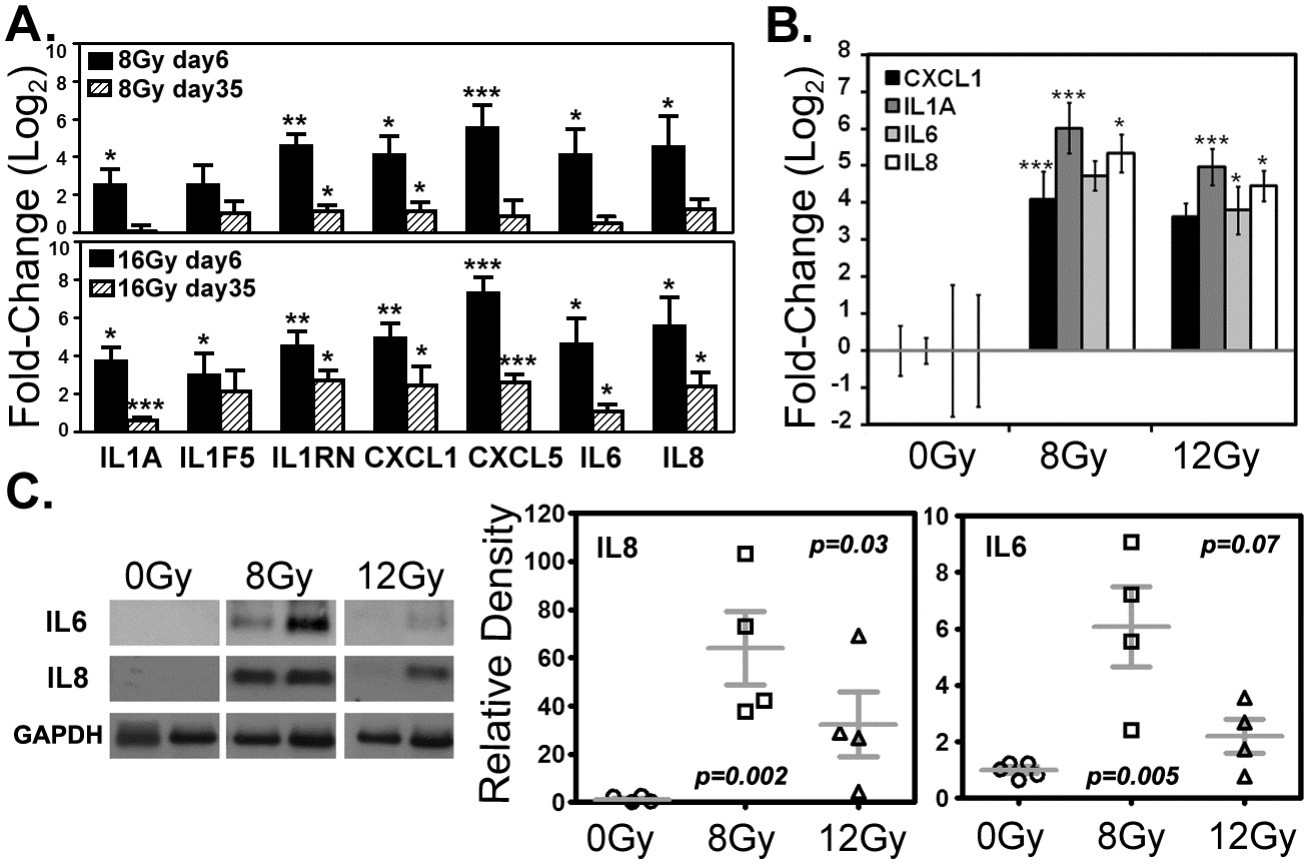
Selected gene expression by quantitative PCR and protein levels by western blot. (A.) Short- and long-term expression of IL1A, IL1F5, IL1RN, CXCL1, CXCL5, IL6 and IL8 detected in U87-MG cell populations *in vitro* at days 6 and 35, after doses of 8 or 16Gy. (B.) Expression of CXCL1, IL1A, IL6, and IL8 in “recurrent” tumor xenografts weeks after tumor formation *in vivo*. SD is shown. (A. & B.) SD is shown, *p < 0.05, **p < 0.01, ***p < 0.005. (C.) Representative western blot and protein quantification for IL6 and IL8 in recurrent tumor xenograft samples. The mean and SEM is shown.

Important to the overall interpretation of the post-irradiation story were the expression results from cytokine and chemokine families, validated by quantitative PCR. These included *IL1* family members *IL1A*, a pleiotropic cytokine with high impact on inflammatory processes and immune response, and *IL1* receptor antagonists *IL1F5* and *IL1RN*; well-known inflammatory mediators *IL6* and *IL8*; and chemokines *CXCL1* and *CXCL5*. On days 6 and 35 following irradiation, all but one gene, *IL1F5*, were found to be significantly up-regulated in 16Gy samples (Fig 3A). Not only was this long-term response measureable in cell culture, but “recurrent” xenograft tumors that did not develop until several weeks after 8 or 12Gy irradiation also displayed sustained increases in gene expression for *IL1A*, *CXCL1*, *IL6* and *IL8* (Fig 3B). Western blots confirmed significant increases in *IL6* and *IL8* protein levels in these “recurrent” tumor xenografts for the experimental points (Fig 3C). This is particularly noteworthy given the extended period between irradiation, tumor formation and subsequent sacrifice (Fig 1E). These results strongly suggest that lasting alterations, driven by the signaling of the irradiated cancer cells, produce an inflammatory milieu in the recurrent tumor microenvironment that is preserved in the *in vivo* setting.

### Computational modeling points to global host inhibitory factors over localized cellular inhibition that slows tumor regrowth

**Fig 4:**
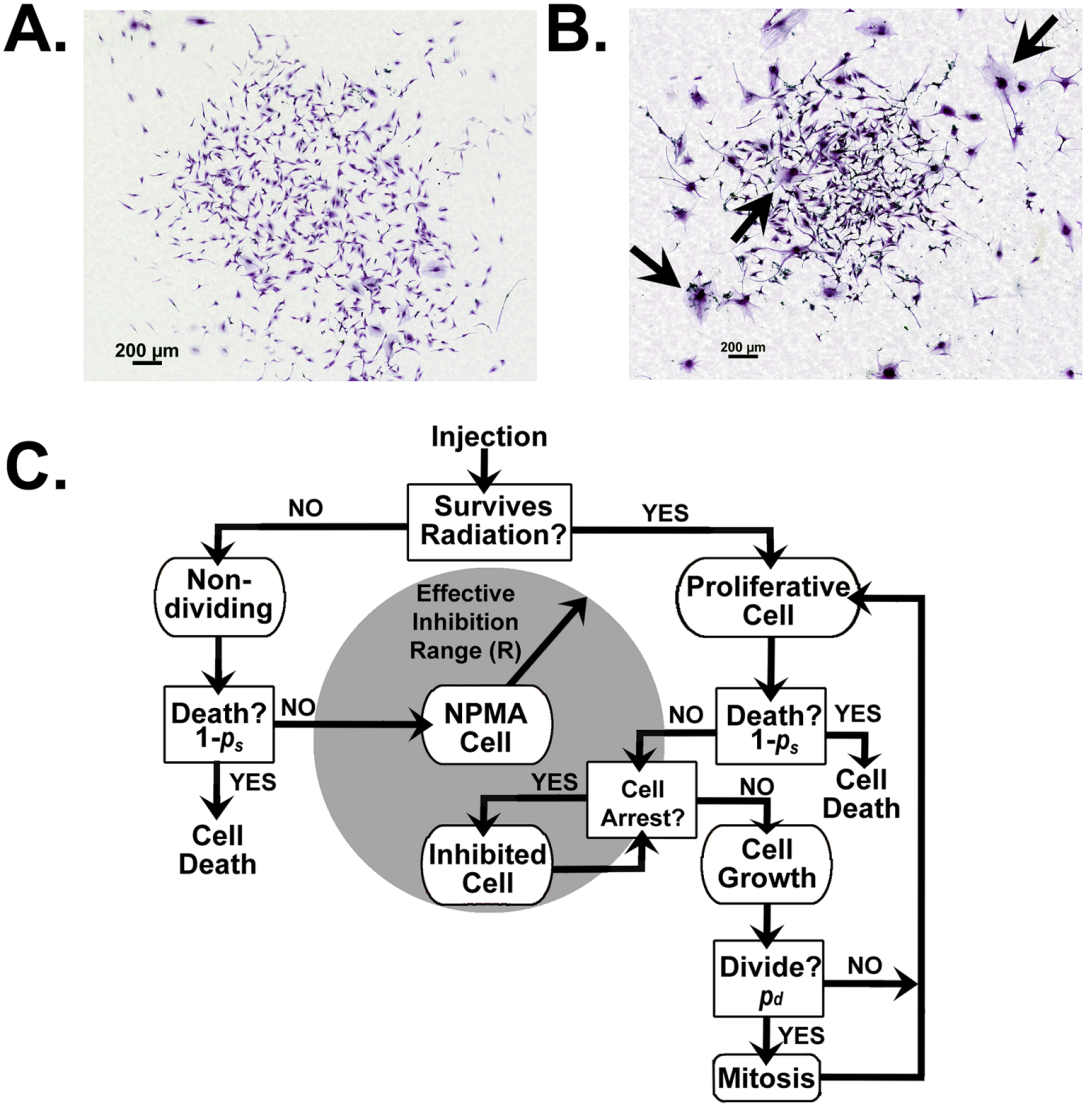
Cellular automaton modeling of post-irradiation tumor growth. Representative colonies stained with crystal violet following (A.) 0Gy or (B.) 16Gy irradiation. Arrows highlight the appearance of the phenotypically-recognizable NPMA cell subtype here considered (C.) Cellular automaton model flowchart for independent decision making by NMPA, proliferating or quiescent cells parameterized by the U87-MG cell line.

With >8Gy exposure, over 96% of the cell population does not maintain clonogenic viability and yet these, destined to die cells, account for the bulk tumor mass until a time when those less than 4% of clonogenically viable cells (presumably including cancer stem cells) proliferate and ultimately repopulate the tumor. Phenotypically, examples indicative of these non-proliferative but metabolically active (NPMA) cells that do not undergo cell death (*e*.*g*. mitotic catastrophe, apoptosis, necrosis, *etc*.) can be seen as giant flattened cells within the *in vitro* population (Fig 4A and 4B) and are characteristic of the SASP [19–21, 23]. Within the first 2 weeks after irradiation, these cells would be expected to have a significant influence on promoting or inhibiting tumor extinction by secreting various inflammatory factors [24]. If the robust inflammatory signature observed at 4 and 6 days following irradiation were exclusively from this population, it would explain the loss of this signature *in vitro* by 35 days after exposure when proliferative progeny account for the majority population.

**Fig 5:**
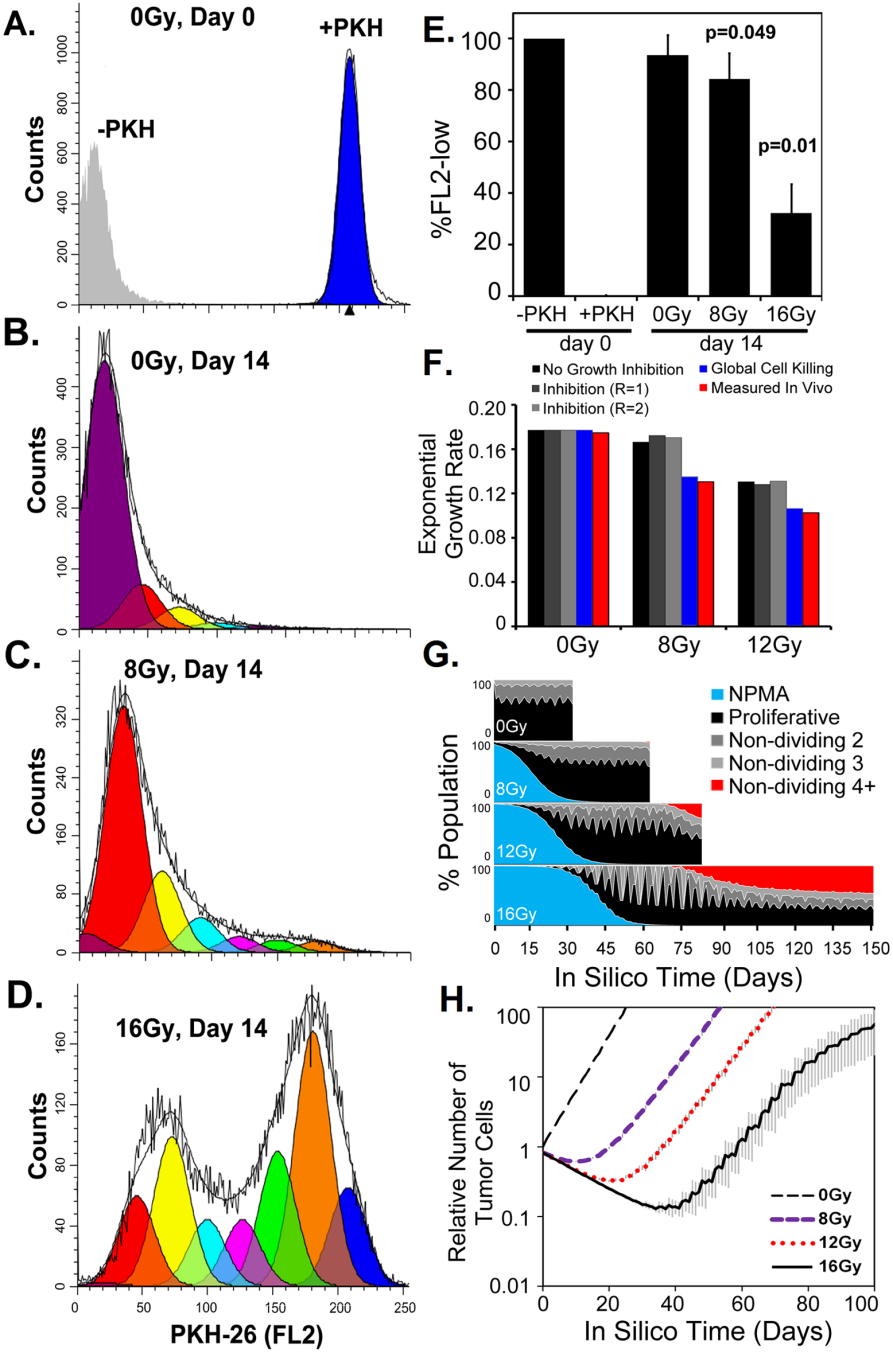
Tracking cell divisions post-irradiation for the cellular automaton computational modeling. (A-D.) Representative flow cytometry analysis of the number of cellular divisions after irradiation in the *in vitro* colony formation assay. Using the ModFit LT3.2 software, raw data (black lines) were fit and modeled for proliferation that results in decreased PKH-26 fluorescent intensity. The number of divisions (1 = orange, 2 = green, 3 = pink, etc.) from the (A) initial untreated day 0 cells with PKH-26 stain (blue) or without (grey) were measured at day 14 following (B.) 0Gy, (C.) 8Gy or (D.) 16Gy irradiation. (E.) Quantification of cells maintaining reproductive integrity were measured as cells undergoing two or more cellular divisions thus decreasing the PKH-26 fluorescent intensity (FL2-low). N = 3, SD is shown, *p-values* were compared to 0Gy day 14. (C.) Exponential growth rates measured in 10 independent simulations for inhibitory conditions compared to *in vivo* observations. (D.) Cellular tracking *in silico* of NPMA or proliferative cells. The proliferative population was also divided by monitoring those cells that have not divided for 2, 3 or 4 or more time steps. (E.) Cell growth *in silico* recapitulates the observed tumor growth rates. N = 3, SD is shown.

To address and further clarify the drivers of tumor extinction versus regrowth dynamics *in vivo*, we used a quantitative, cellular automaton, tumor growth model including parameterization of the NPMA cell population (Fig 4C). To quantify this population, U87-MG cells were labeled with the lipophilic dye PKH-26 to track cellular division following irradiation in the *in vitro* clonogenic survival assay. (Fig 5A–5D). Fourteen-days following radiation exposure, a significant decrease in proliferating cells was observed with 8 and 16Gy exposure and the percent of NPMA cells (PKH-26^high^) was estimated (Fig 5E). Using this data, the NPMA post-irradiation population was modeled to determine if it could be a source of inhibitory factors sufficient to provide a match to the observed growth rates using predictions from the cellular automaton model of tumor growth.

First, only cell death due to irradiation without any NPMA sup-population was considered, which was, in itself, sufficient to result in a delay in tumor formation, as was experimentally observed, but nevertheless then gave rise to substantially higher tumor growth rates than what was measured *in vivo* (Figs 1F and 5F). This disparity demonstrated an additional inhibitory effect must be involved in suppression of tumor “regrowth” from the post-irradiated cell population. Next, we assumed the factors secreted by the NPMA cells could locally inhibit cellular growth within an effective range (*R = 1* denotes a cell diameter length). Our simulation results show that even an additional local inhibition from NPMA cells (up to a two-cell diameter range, R = 2) would, somewhat surprisingly, still have very little modulating effect on overall tumor growth rates. This theoretical observation suggested the involvement of a host systematic effect is required for the suppression of the tumor expansion experimentally observed. Finally, we tested the assumption that the NPMA cells could induce a systemic increase in tumor cell kill by the host, for instance via triggering the immune system. Now, when the cellular death rate probability (*p*_*a*_) is increased to 5.5% post-8Gy and 6% post-12Gy, this global inhibition resulted in reduced overall tumor growth dynamics to identically match that observed by the *in vivo* measurements and also yielded survival times that more closely matched the measured survival time (Fig 5F). Interestingly, as the radiation dose increased, the number of cells which did not divide for 4 or more time steps increased to nearly 50% of the total population (Fig 5G and 5H). The consequence of these results indicates such recurrent tumors will be more resistant to subsequent tumor irradiation, which has a higher effectiveness on actively proliferating targets, due to overall kinetic shifts in the population.

## Discussion

A focused examination of the time-dependent, perturbation of the cancer cell population, from early to long times after irradiation, is critical for understanding the potential for post-therapy regrowth and treatment failure of GBM. Yet, studies of the long-term radiation responses in GBM cell populations have been largely neglected. Here, measured cellular, kinetic and molecular responses of GBM cell populations, as detected several days after irradiation, were complemented by data obtained weeks after irradiation, at which time the population was heavily composed of progeny from the reproductively-viable cells that survived the cytotoxic treatment. Furthermore, the additional selective survival pressures of GBM regrowth under the constraints of *in vivo* influence were identified and investigated using a xenograft model in which cancer cells were irradiated *ex vivo*. Both the *in vitro* and *in vivo* systems revealed a robust expression of inflammatory genes over days to months after irradiation. But, surprisingly, only in the *in vivo* case were the GBM growth dynamics significantly inhibited at long times after irradiation. Through use of a cellular automaton model of tumor growth that allowed the testing of potential sources of inhibitory factors, only a global (systemic) tumor inhibition was found to be critical to modulating the eventual regrowth capacity of the surviving tumor cells. This data suggests the *ex vivo* irradiated tumor cells elicit a substantial response from the non-irradiated host tissues that alters the capacity for tumor regrowth.

Following high dose, low dose-rate irradiation, previous experiments have observed a lack of change as well as alteration to intrinsic tumor growth dynamics in U188MG and U373MG, respectively [25]. Cell recovery, following exposure to high doses of IR, activates canonical pathways similar to what is seen following exposure to generalized damaging agents such as pathogens and chemicals. Irradiation of normal brain tissue typically initiates a rapid inflammatory spiked response, acting through chemokines and pro-inflammatory cytokines, which is soon diminished [26]. However, beyond the classic cell cycle arrest and DNA repair resolution in surviving irradiated cells, this signal can be aberrantly reactivated, weeks and months or even years after exposure, which may be interpreted as failed attempts to complete the wound healing process [27]. These extracellular factors signal stress to the surrounding tissue in an attempt to orchestrate cell population and tissue repair, but perhaps also providing a mechanism towards increased tumor aggressiveness with enhanced treatment resistance. This persistent inflammatory process has potential to mediate radiation-associated bystander effects and result in propagated cell and tissue damage.

There is strong evidence that inflammatory mediators play a critical role in the pathogenesis of GBM and may provide novel targets to improve therapeutic intervention [28–30]. Fueling this argument are the findings of these studies following irradiation of GBM cell populations that showed a significant increase in multiple chemokines and inflammatory cytokines, particularly interleukins. The strong activation of this inflammatory response serves overlapping positive and negative roles in both normal tissue recovery and recurrent tumor evolution. The unique experimental conditions utilized in this study, *ex vivo* cancer cell irradiation and *in vivo* implantation without normal tissue irradiation, highlight the preserved shift in the tumor phenotype away from the original pre-treatment growth resulting in an altered persistent inflammatory environment. That approximately 50% of the *in vivo* implantations of irradiated cell populations were driven to extinction (4 of 8 with 8Gy and 4 of 7 with 12Gy), in contrast to the *in vitro* case, indicates the host’s ability to elicit a reciprocal suppressive response to the *in vivo* implantation. This *in vivo* systemic inhibition of irradiated cells is also reflected in the fact that even when the irradiated cells successfully formed tumors, the tumors exhibited reduced growth rates, never obtaining their pre-irradiated growth rate. This is in contrast to the fact these irradiated GBM cells when grown *in vitro* demonstrate an ability to return to pre-irradiated growth rates with no alteration in long-term population doublings. Computational modeling of this inhibitory effect determined that a localized inhibition by the irradiated cancer cells themselves could not explain this reduction in growth rate. Introduction of modest death rates (4% and 7% at 8 and 12Gy respectively) on the systemic scale suggests that manipulation of the host-tumor interaction at 1–3 weeks following irradiation, before the cell population’s spontaneous restoration to pre-irradiation population doubling rates as observed *in vitro*, may offer a previously unexploited window and novel opportunity for treatment intervention. Importantly, a limitation of our xenograft model is the absence of an adaptive immune system in the nude mice used here. While we expect IR to still elicit an inflammatory response, the subsequent reaction of a complete central nervous system with an immune privileged environment is not possibly examined in our current model. These studies suggest, further work is justified under *in vivo* orthotropic conditions of a syngeneic immunocompetent host with or without agents that can modify the inflammatory mediators of potential recurrent tumor formation following the delivery highly cytotoxic radiation treatment.

Recently, an integrative analysis, utilizing a number of discrete data types (e.g. mRNA expression microarray, copy number variation data, methylation microarray) from the cancer genome atlas (TCGA) project, was performed as a comprehensive approach to analyze 248 GBM samples [31]. Uncovered was a striking enrichment of the immune/inflammatory response that correlated negatively with patient survival time. Improved understanding of the functional reach of these dysregulated inflammatory genes, within the context of radiation intervention, may expose novel therapeutic candidates which surface while the tumor population is recovering from the cytotoxic insult and is thus transiently venerable to targeted intervention of these signalings. As a case in point, blocking the TGF-beta type I receptor kinase before IR exposure has been shown to decrease glioma-initiating cell proliferation and radiation resistance as well as delayed *in vivo* glioblastoma tumor growth in murine models thereby increasing survival [32, 33].

The altered molecular pathways observed here begin to reveal, in a time-dependent manner, how the radiation-naïve GBM cell population is perturbed in response to the cytotoxic insult of high doses of IR. The strong up-regulation of genes, particularly of inflammatory genes, in the days after irradiation gives an indication of not only change within the cancer cells themselves but the potential impact of such irradiated cells on the surrounding tumor milieu. Although, following high-dose irradiation the vast majority of the GBM cells within the tumor population will ultimately become reproductively non-viable, the impact of these non-viable cells greatly alters the tumor milieu present during the early post-irradiation growth of those few reproductively viable cancer cells and of their progeny. Thus, post-irradiation cell signaling, including from those cells destined to die, significantly impacts the inevitable resurgence of the population, thereby setting the course for any recurrence. An understanding of the time-dependent, signaling mechanisms arising from the post-irradiated population, which modulate the escape from extinction of the surviving cancer cells, is paramount to improving therapeutic response.

Current clinical trials attempting to overcome acquired treatment resistance of recurrent GBM through targeted therapies are built upon a standard of care with surgery, radiotherapy and chemotherapy. However, this aggressive multimodality therapy no doubt induces treatment-related genetic and epigenetic alterations in the surviving cancer cells that will shape the evolution of the recurrent tumor, alter the surrounding microenvironment and modulate the global host response [34]. While new targeted therapies have well-characterized mechanisms of action, pre-clinical studies must account for molecular shifts away from the initial heterogeneous pre-treatment GBM cell population and taking into account therapy-induced tumor evolution and the accompanying emergence of new and altered targets. This study focuses on the impact of high doses of radiation that push the tumor population to near extinction, thereby sculpting the molecular fingerprint and dynamics of the re-emergent tumor population. In these studies the temporal rebound of the irradiated populations revealed a common inflammatory signature observed *in vitro* as well as in the *in vivo* tumors derived from injected GBM populations receiving high dose radiation. Additional comparisons to previously measured tumor bed effects of irradiating normal tissue might shed light on the vulnerabilities of a remerging tumor. Pre-clinical strategies will benefit from inclusion of models of such rebounding tumor populations, not only to account for common molecular shifts in the tumors themselves, but to gauge the time-dependent systemic response of the host, to better inform therapeutic decision making and thereby ultimately increase therapeutic efficacy.

## Supporting information

S1 Table Illumina gene expression results. Microarray results of 281 statistically significant genes differentially regulated between 1, 4, 6 or 35 days after 8Gy or 16Gy (FDR < 10% and fold change > ±1.5).
(PDF)

S2 Table GSEA gene ontology analysis. Statistically significant (FWER < 5%) gene sets up- or down-regulated compared to 0Gy.
(PDF)

S3 Table Functional gene classification using DAVID. The top three enrichment groups found from the 273 identifiable genes with statistical significance.
(PDF)
