# Supplementary material for: Host mediated inflammatory influence on glioblastoma multiforme recurrence following high-dose ionizing radiation
Source: PLoS One. 2017 May 22;12(5):e0178155. doi: 10.1371/journal.pone.0178155 (PMC5439715; doi:10.1371/journal.pone.0178155)
Supplement: S1 Table — Microarray results of 281 statistically significant genes differentially regulated between 1, 4, 6 or 35 days after 8Gy or 16Gy (FDR < 10% and fold change > ±1.5). (PDF) [file pone.0178155.s001.pdf]

**S1 Table. Illumina gene expression results.** Microarray results of 281 statistically significant genes differentially regulated between 1, 4, 6 or 35 days after 8Gy or 16Gy (FDR < 10% and fold change > ±1.5).

| Name | Description | Entrez ID | ANOVA p-value | FDR (Bonferroni-Holm) |
|---|---|---|---|---|
| IL1RN | interleukin 1 receptor antagonist | 3557 | 2.22E-16 | 6.95E-12 |
| CDC42EP4 | CDC42 effector protein (Rho GTPase binding) 4 | 23580 | 4.44E-16 | 1.39E-11 |
| CXCL5 | chemokine (C-X-C motif) ligand 5 | 6374 | 6.66E-16 | 2.09E-11 |
| HIST2H2BE | histone cluster 2, H2be | 8349 | 1.11E-15 | 3.48E-11 |
| ATF3 | activating transcription factor 3 | 467 | 1.50E-14 | 4.69E-10 |
| PSG4 | pregnancy specific beta-1-glycoprotein 4 | 5672 | 2.02E-14 | 6.33E-10 |
| CCL3L3 | chemokine (C-C motif) ligand 3-like 3 | 414062 | 2.07E-14 | 6.46E-10 |
| CCL3 | chemokine (C-C motif) ligand 3 | 6348 | 3.23E-14 | 1.01E-09 |
| CCL3L1 | chemokine (C-C motif) ligand 3-like 1 | 6349 | 4.60E-14 | 1.44E-09 |
| CCL20 | chemokine (C-C motif) ligand 20 | 6364 | 5.25E-14 | 1.64E-09 |
| CSF2 | colony stimulating factor 2 (granulocyte-macrophage) | 1437 | 6.32E-14 | 1.98E-09 |
| MMP12 | matrix metallopeptidase 12 (macrophage elastase) | 4321 | 8.42E-14 | 2.63E-09 |
| IL24 | interleukin 24 | 11009 | 9.03E-14 | 2.82E-09 |
| DCN | decorin | 1634 | 1.11E-13 | 3.48E-09 |
| CXCL1 | chemokine (C-X-C motif) ligand 1 | 2919 | 1.15E-13 | 3.61E-09 |
| DPYSL3 | dihydropyrimidinase-like 3 | 1809 | 2.35E-13 | 7.37E-09 |
| AURKB | aurora kinase B | 9212 | 2.41E-13 | 7.55E-09 |
| CHI3L2 | chitinase 3-like 2 | 1117 | 2.66E-13 | 8.31E-09 |
| CCNB2 | cyclin B2 | 9133 | 4.68E-13 | 1.46E-08 |
| MMP10 | matrix metallopeptidase 10 | 4319 | 5.37E-13 | 1.68E-08 |
| IL6 | interleukin 6 | 3569 | 7.43E-13 | 2.32E-08 |
| IL1F5 | interleukin 1 family, member 5 (delta) | 26525 | 1.12E-12 | 3.49E-08 |
| SPRR2D | small proline-rich protein 2D | 6703 | 1.21E-12 | 3.79E-08 |
| CDKN1A | cyclin-dependent kinase inhibitor 1A (p21, Cip1) | 1026 | 1.60E-12 | 4.99E-08 |
| CCL5 | chemokine (C-C motif) ligand 5 | 6352 | 1.61E-12 | 5.04E-08 |
| LOC387763 | hypothetical | 387763 | 1.64E-12 | 5.13E-08 |
| PI3 | peptidase inhibitor 3 | 5266 | 2.00E-12 | 6.23E-08 |
| PTGS2 | prostaglandin-endoperoxide synthase 2 | 5743 | 2.11E-12 | 6.60E-08 |
| SLC25A24 | solute carrier family 25 (mitochondrial carrier) | 29957 | 2.13E-12 | 6.65E-08 |
| DEFB103B | defensin, beta 103B | 414325 | 2.17E-12 | 6.77E-08 |
| IL1F10 | interleukin 1 family, member 10 (theta) | 84639 | 2.30E-12 | 7.18E-08 |
| KIFC1 | kinesin family member C1 | 3833 | 2.96E-12 | 9.23E-08 |
| KIF20A | kinesin family member 20A | 10112 | 3.41E-12 | 1.07E-07 |
| FOSL1 | FOS-like antigen 1 | 8061 | 4.50E-12 | 1.40E-07 |
| PITPNC1 | phosphatidylinositol transfer protein, cytoplasmic 1 | 26207 | 6.09E-12 | 1.90E-07 |
| FLJ16165 | purple acid phosphatase long form | 390928 | 8.78E-12 | 2.74E-07 |
| CXCL2 | chemokine (C-X-C motif) ligand 2 | 2920 | 1.06E-11 | 3.32E-07 |
| MAFB | v-maf musculoaponeurotic fibrosarcoma oncogene homolog B | 9935 | 1.18E-11 | 3.69E-07 |
| IL1A | interleukin 1, alpha | 3552 | 1.22E-11 | 3.79E-07 |
| SERTAD1 | SERTA domain containing 1 | 29950 | 1.74E-11 | 5.42E-07 |
| LIF | leukemia inhibitory factor | 3976 | 2.05E-11 | 6.37E-07 |
| LRRC15 | leucine rich repeat containing 15 | 131578 | 2.17E-11 | 6.77E-07 |
| HIST1H2BD | histone cluster 1, H2bd | 3017 | 2.22E-11 | 6.90E-07 |
| FCRLA | Fc receptor-like A | 84824 | 2.42E-11 | 7.52E-07 |
| CDCA8 | cell division cycle associated 8 | 55143 | 2.60E-11 | 8.11E-07 |
| C20ORF100 | chromosome 20 open reading frame 100 | 84969 | 2.68E-11 | 8.34E-07 |
| IL1F8 | interleukin 1 family, member 8 (eta) | 27177 | 2.68E-11 | 8.34E-07 |
| ALDH7A1 | aldehyde dehydrogenase 7 family, member A1 | 501 | 3.39E-11 | 1.05E-06 |
| SCG2 | secretogranin II (chromogranin C) | 7857 | 3.62E-11 | 1.13E-06 |

| | | | | |
|---|---|---|---|---|
| ERN1 | endoplasmic reticulum to nucleusing 1 | 2081 | 3.77E-11 | 1.17E-06 |
| ADORA2A | adenosine A2a receptor | 135 | 3.81E-11 | 1.18E-06 |
| HIST2H2AA3 | histone cluster 2, H2aa3 | 8337 | 4.50E-11 | 1.40E-06 |
| TOX2 | TOX high mobility group box family member 2 | 84969 | 5.25E-11 | 1.63E-06 |
| DUSP1 | dual specificity phosphatase 1 | 1843 | 9.04E-11 | 2.81E-06 |
| IL12A | interleukin 12A (natural killer cell stimulatory factor 1) | 3592 | 9.24E-11 | 2.87E-06 |
| LCP1 | lymphocyte cytosolic protein 1 (L-plastin) | 3936 | 9.33E-11 | 2.90E-06 |
| LOC100133511 | PREDICTED: similar to complement component C3 | 100133511 | 1.02E-10 | 3.17E-06 |
| SERPINB4 | serpin peptidase inhibitor, clade B member 4 | 6318 | 1.07E-10 | 3.34E-06 |
| NCAPD2 | non-SMC condensin I complex, subunit D2 | 9918 | 1.12E-10 | 3.47E-06 |
| SPINK1 | serine peptidase inhibitor, Kazal type 1 | 6690 | 1.23E-10 | 3.82E-06 |
| SOD2 | superoxide dismutase 2, mitochondrial | 6648 | 1.28E-10 | 3.96E-06 |
| PCLO | piccolo (presynaptic cytomatrix protein) | 27445 | 1.30E-10 | 4.04E-06 |
| MGC10997 | pseudogene MGC10997 on chromosome 15. | 84741 | 1.59E-10 | 4.92E-06 |
| ESM1 | endothelial cell-specific molecule 1 | 11082 | 1.61E-10 | 5.00E-06 |
| TNFAIP3 | tumor necrosis factor, alpha-induced protein 3 | 7128 | 2.59E-10 | 8.02E-06 |
| CCNA2 | cyclin A2 | 890 | 2.80E-10 | 8.66E-06 |
| LOC346887 | PREDICTED: similar to solute carrier family 16 | 346887 | 2.89E-10 | 8.94E-06 |
| FOSB | FBJ murine osteosarcoma viral oncogene homolog B | 2354 | 2.99E-10 | 9.26E-06 |
| HIST2H2AA4 | histone cluster 2, H2aa4 | 723790 | 3.00E-10 | 9.28E-06 |
| FST | follistatin | 10468 | 3.07E-10 | 9.52E-06 |
| SLC2A6 | solute carrier family 2 member 6 | 11182 | 3.32E-10 | 1.03E-05 |
| NAV1 | neuron navigator 1 | 89796 | 3.40E-10 | 1.05E-05 |
| CYP26B1 | cytochrome P450, family 26, subfamily B | 56603 | 3.49E-10 | 1.08E-05 |
| LOC653879 | PREDICTED: similar to Complement C3 precursor | 653879 | 3.57E-10 | 1.11E-05 |
| DUSP10 | dual specificity phosphatase 10 | 11221 | 3.73E-10 | 1.15E-05 |
| IL1F9 | interleukin 1 family, member 9 | 56300 | 4.03E-10 | 1.25E-05 |
| HIST1H4H | histone cluster 1, H4h | 8365 | 4.11E-10 | 1.27E-05 |
| ABI3BP | ABI gene family, member 3 binding protein | 25890 | 4.38E-10 | 1.35E-05 |
| BAMBI | BMP and activin membrane-bound inhibitor homolog | 25805 | 4.78E-10 | 1.48E-05 |
| SAT1 | spermidine/spermine N1-acetyltransferase 1 | 6303 | 4.80E-10 | 1.49E-05 |
| CLCF1 | cardiotrophin-like cytokine factor 1 | 23529 | 4.84E-10 | 1.50E-05 |
| HIST1H2BG | histone cluster 1, H2bg (HIST1H2BG) | 8339 | 5.10E-10 | 1.58E-05 |
| DIO2 | deiodinase, iodothyronine, type II | 1734 | 5.24E-10 | 1.62E-05 |
| MAFF | v-maf musculoaponeurotic fibrosarcoma oncogene homolog F | 23764 | 5.60E-10 | 1.73E-05 |
| EEF1D | eukaryotic translation elongation factor 1 delta | 1936 | 5.89E-10 | 1.82E-05 |
| TXNIP | thioredoxin interacting protein | 10628 | 6.38E-10 | 1.97E-05 |
| CTPS2 | CTP synthase II | 56474 | 6.54E-10 | 2.02E-05 |
| SLC16A6 | solute carrier family 16, member 6 | 9120 | 6.83E-10 | 2.11E-05 |
| IMPDH2 | IMP (inosine monophosphate) dehydrogenase 2 | 3615 | 7.44E-10 | 2.30E-05 |
| CXXC5 | CXXC finger 5 | 51523 | 9.09E-10 | 2.80E-05 |
| CPS1 | carbamoyl-phosphate synthetase 1, mitochondrial | 1373 | 9.10E-10 | 2.81E-05 |
| LUM | lumican | 4060 | 9.71E-10 | 2.99E-05 |
| C5ORF13 | chromosome 5 open reading frame 13 | 9315 | 1.02E-09 | 3.16E-05 |
| LOC731314 | PREDICTED: similar to H2A histone family, member X | 731314 | 1.04E-09 | 3.21E-05 |
| SMTN | smoothelin | 6525 | 1.15E-09 | 3.56E-05 |
| CSF3 | colony stimulating factor 3 (granulocyte) | 1440 | 1.35E-09 | 4.17E-05 |
| GADD45B | growth arrest and DNA-damage-inducible, beta | 4616 | 1.43E-09 | 4.39E-05 |
| NFKBIZ | nuclear factor kappa light B-cell enhancer inhibitor, zeta | 64332 | 1.51E-09 | 4.65E-05 |
| NRG1 | neuregulin 1 | 3084 | 1.53E-09 | 4.70E-05 |
| ANKRD1 | ankyrin repeat domain 1 | 27063 | 1.68E-09 | 5.17E-05 |
| HIST1H4C | histone cluster 1, H4c | 8364 | 2.54E-09 | 7.80E-05 |
| CYP4B1 | cytochrome P450, family 4, subfamily B, polypeptide 1 | 1580 | 2.62E-09 | 8.03E-05 |
| LOXL4 | lysyl oxidase-like 4 | 84171 | 3.28E-09 | 1.01E-04 |
| HIST1H2AC | histone cluster 1, H2ac | 8334 | 3.37E-09 | 1.03E-04 |
| LOC654433 | hypothetical LOC654433 | 654433 | 3.41E-09 | 1.04E-04 |
| C3 | complement component 3 | 718 | 3.60E-09 | 1.10E-04 |
| DTNA | dystrobrevin, alpha | 1837 | 3.67E-09 | 1.13E-04 |
| HJURP | Holliday junction recognition protein | 55355 | 3.77E-09 | 1.15E-04 |

| | | | | |
|---|---|---|---|---|
| IER3 | immediate early response 3 | 8870 | 3.90E-09 | 1.19E-04 |
| HSD11B1 | hydroxysteroid (11-beta) dehydrogenase 1 | 3290 | 4.03E-09 | 1.23E-04 |
| DUSP5 | dual specificity phosphatase 5 | 1847 | 4.09E-09 | 1.25E-04 |
| HAS1 | hyaluronan synthase 1 | 3036 | 4.54E-09 | 1.39E-04 |
| DDIT3 | DNA-damage-inducible transcript 3 | 1649 | 4.88E-09 | 1.49E-04 |
| MARCH4 | membrane-associated ring finger (C3HC4) | 57574 | 4.92E-09 | 1.50E-04 |
| HMGCS1 | 3-hydroxy-3-methylglutaryl-Coenzyme A synthase 1 | 3157 | 5.70E-09 | 1.74E-04 |
| SARS2 | seryl-tRNA synthetase 2, mitochondrial | 54938 | 6.05E-09 | 1.85E-04 |
| DDIT4L | DNA-damage-inducible transcript 4-like | 115265 | 6.29E-09 | 1.92E-04 |
| IL8 | interleukin 8 | 3576 | 6.33E-09 | 1.93E-04 |
| DKK1 | dickkopf homolog 1 | 22943 | 6.70E-09 | 2.05E-04 |
| RND3 | Rho family GTPase 3 | 390 | 6.90E-09 | 2.11E-04 |
| ANKRD13A | ankyrin repeat domain 13A | 88455 | 7.16E-09 | 2.18E-04 |
| RCAN1 | regulator of calcineurin 1 | 1827 | 7.44E-09 | 2.27E-04 |
| FHOD1 | formin homology 2 domain containing 1 | 29109 | 7.62E-09 | 2.32E-04 |
| SERPINB2 | serpin peptidase inhibitor, clade B | 5055 | 7.86E-09 | 2.40E-04 |
| ALDH3B1 | aldehyde dehydrogenase 3 family, member B1 | 221 | 7.95E-09 | 2.42E-04 |
| TNFRSF10B | tumor necrosis factor receptor superfamily, member 10b | 8795 | 8.15E-09 | 2.49E-04 |
| ITGAX | integrin, alpha X (complement component 3 receptor 4) | 3687 | 8.36E-09 | 2.55E-04 |
| MIR302C | microRNA 302c | 442895 | 9.04E-09 | 2.75E-04 |
| AKR1C4 | aldo-keto reductase family 1, member C4 | 1109 | 9.22E-09 | 2.81E-04 |
| IL33 | interleukin 33 | 90865 | 9.29E-09 | 2.83E-04 |
| TUBGCP2 | tubulin, gamma complex associated protein 2 | 10844 | 9.31E-09 | 2.84E-04 |
| ZNF503 | zinc finger protein 503 | 84858 | 9.53E-09 | 2.90E-04 |
| FLJ22639 | PREDICTED: hypothetical protein | 79854 | 9.66E-09 | 2.94E-04 |
| PTGES | prostaglandin E synthase | 9536 | 9.89E-09 | 3.01E-04 |
| SLC39A14 | solute carrier family 39 | 23516 | 1.04E-08 | 3.18E-04 |
| DAB2 | disabled homolog 2, mitogen-responsive phosphoprotein | 1601 | 1.06E-08 | 3.24E-04 |
| MIR1974 | microRNA 1974 | 100302207 | 1.20E-08 | 3.66E-04 |
| RASD1 | RAS, dexamethasone-induced 1 | 51655 | 1.29E-08 | 3.92E-04 |
| HBEGF | heparin-binding EGF-like growth factor | 1839 | 1.46E-08 | 4.45E-04 |
| LOC100129104 | PREDICTED: similar hydroxyproline-rich glycoprotein VSP-3 | 100129104 | 1.53E-08 | 4.65E-04 |
| TNFRSF14 | tumor necrosis factor receptor superfamily, member 14 | 8764 | 1.55E-08 | 4.71E-04 |
| AHNAK2 | AHNAK nucleoprotein 2 | 113146 | 1.63E-08 | 4.95E-04 |
| MIR1275 | microRNA 1275 | 100302123 | 1.75E-08 | 5.31E-04 |
| ZNF385D | zinc finger protein 385D | 79750 | 1.79E-08 | 5.42E-04 |
| ZNF175 | zinc finger protein 175 | 7728 | 1.89E-08 | 5.74E-04 |
| XPOT | exportin, tRNA | 11260 | 2.06E-08 | 6.23E-04 |
| HSPA6 | heat shock 70kDa protein 6 | 3310 | 2.38E-08 | 7.19E-04 |
| IL11 | interleukin 11 | 3589 | 2.41E-08 | 7.28E-04 |
| CRYM | crystallin, mu | 1428 | 2.50E-08 | 7.56E-04 |
| NRGN | neurogranin (protein kinase C substrate, RC3) | 4900 | 2.52E-08 | 7.61E-04 |
| CARD10 | caspase recruitment domain family, member 10 | 29775 | 2.76E-08 | 8.35E-04 |
| TSPYL2 | PREDICTED: TSPY-like 2, transcript variant 3 (TSPYL2) | 64061 | 2.79E-08 | 8.42E-04 |
| SGK1 | serum/glucocorticoid regulated kinase 1 | 6446 | 2.89E-08 | 8.74E-04 |
| RIMS3 | regulating synaptic membrane exocytosis 3 | 9783 | 3.09E-08 | 9.33E-04 |
| PARM1 | prostate androgen-regulated mucin-like protein 1 | 25849 | 3.34E-08 | 1.01E-03 |
| FOLR3 | folate receptor 3 (gamma) | 2352 | 3.45E-08 | 1.04E-03 |
| KIAA0367 | KIAA0367 | 23273 | 3.89E-08 | 1.17E-03 |
| GADD45A | growth arrest and DNA-damage-inducible, alpha | 1647 | 3.99E-08 | 1.20E-03 |
| TNFAIP6 | tumor necrosis factor, alpha-induced protein 6 | 7130 | 4.12E-08 | 1.24E-03 |
| ENO2 | enolase 2 (gamma, neuronal) | 2026 | 4.47E-08 | 1.34E-03 |
| ST3GAL1 | ST3 beta-galactoside alpha-2,3-sialyltransferase 1 | 6482 | 4.59E-08 | 1.38E-03 |
| ARHGDIB | Rho GDP dissociation inhibitor (GDI) beta | 397 | 4.60E-08 | 1.38E-03 |
| SVEP1 | sushi, von Willebrand factor type A, EGF and pentraxin domain containing 1 | 79987 | 4.64E-08 | 1.39E-03 |
| ADM | adrenomedullin | 133 | 4.90E-08 | 1.47E-03 |
| DDR2 | discoidin domain receptor tyrosine kinase 2 | 4921 | 4.96E-08 | 1.49E-03 |
| LOC100132805 | PREDICTED: similar to predicted protein | 100132805 | 5.04E-08 | 1.51E-03 |

| | | | | |
|---|---|---|---|---|
| ANGPTL4 | angiopoietin-like 4 | 51129 | 5.10E-08 | 1.53E-03 |
| PLD6 | phospholipase D family, member 6 | 201164 | 5.15E-08 | 1.55E-03 |
| ACTA2 | actin, alpha 2, smooth muscle, aorta | 59 | 5.16E-08 | 1.55E-03 |
| CCL2 | chemokine (C-C motif) ligand 2 | 6347 | 5.31E-08 | 1.59E-03 |
| SERPINB8 | serpin peptidase inhibitor, clade B, member 8 | 5271 | 5.72E-08 | 1.72E-03 |
| BMP2 | bone morphogenetic protein 2 | 650 | 6.32E-08 | 1.90E-03 |
| MMP7 | matrix metallopeptidase 7 (matrilysin, uterine) | 4316 | 6.44E-08 | 1.93E-03 |
| SRGN | serglycin | 5552 | 6.72E-08 | 2.01E-03 |
| HAS3 | hyaluronan synthase 3 | 3038 | 6.75E-08 | 2.02E-03 |
| ITPRIP | inositol 1,4,5-triphosphate receptor interacting protein | 85450 | 6.89E-08 | 2.07E-03 |
| RFTN2 | raftlin family member 2 | 130132 | 7.35E-08 | 2.20E-03 |
| JMJD6 | jumonji domain containing 6 | 23210 | 7.52E-08 | 2.25E-03 |
| LOC642567 | PREDICTED: misc_RNA | 642567 | 7.53E-08 | 2.25E-03 |
| ADAMTS1 | ADAM metallopeptidase with thrombospondin type 1 motif | 9510 | 8.05E-08 | 2.41E-03 |
| ACACA | acetyl-Coenzyme A carboxylase alpha | 31 | 8.09E-08 | 2.42E-03 |
| SNORD16 | small nucleolar RNA, C/D box 16 | 595097 | 8.37E-08 | 2.50E-03 |
| MAP3K8 | mitogen-activated protein kinase kinase kinase 8 | 1326 | 8.76E-08 | 2.62E-03 |
| RRAD | Ras-related associated with diabetes | 6236 | 9.42E-08 | 2.81E-03 |
| PCOLCE | procollagen C-endopeptidase enhancer | 5118 | 9.60E-08 | 2.87E-03 |
| LRRN3 | leucine rich repeat neuronal 3 | 54674 | 9.94E-08 | 2.97E-03 |
| PLAUR | plasminogen activator, urokinase receptor | 5329 | 1.01E-07 | 3.02E-03 |
| SGK | serum/glucocorticoid regulated kinase | 6446 | 1.02E-07 | 3.05E-03 |
| DEDD2 | death effector domain containing 2 | 162989 | 1.04E-07 | 3.10E-03 |
| C4ORF7 | chromosome 4 open reading frame 7 | 260436 | 1.11E-07 | 3.30E-03 |
| C1QTNF1 | C1q and tumor necrosis factor related protein 1 | 114897 | 1.14E-07 | 3.41E-03 |
| NQO1 | NAD(P)H dehydrogenase, quinone 1 | 1728 | 1.17E-07 | 3.48E-03 |
| TIMP1 | TIMP metallopeptidase inhibitor 1 | 7076 | 1.22E-07 | 3.63E-03 |
| H1F0 | H1 histone family, member 0 | 3005 | 1.28E-07 | 3.80E-03 |
| LOC650832 | PREDICTED: similar to MAPK kinase 3 isoform A | 650832 | 1.34E-07 | 3.98E-03 |
| TMEM132A | transmembrane protein 132A | 54972 | 1.38E-07 | 4.12E-03 |
| RNU6-15 | RNA, U6 small nuclear 15 | 100302741 | 1.41E-07 | 4.20E-03 |
| UBE2T | ubiquitin-conjugating enzyme E2T | 29089 | 1.57E-07 | 4.66E-03 |
| ETS2 | v-ets erythroblastosis virus E26 oncogene homolog 2 | 2114 | 1.61E-07 | 4.79E-03 |
| LOC338758 | PREDICTED: hypothetical protein LOC338758 | 338758 | 1.68E-07 | 4.99E-03 |
| PPAP2B | phosphatidic acid phosphatase type 2B | 8613 | 1.70E-07 | 5.05E-03 |
| CLDN14 | claudin 14 | 23562 | 1.78E-07 | 5.28E-03 |
| C21ORF7 | chromosome 21 open reading frame 7 | 56911 | 1.82E-07 | 5.38E-03 |
| TRAF3IP2 | TRAF3 interacting protein 2 | 10758 | 1.88E-07 | 5.57E-03 |
| GDF15 | growth differentiation factor 15 | 9518 | 1.92E-07 | 5.68E-03 |
| HIST2H2AC | histone cluster 2, H2ac | 8338 | 2.05E-07 | 6.08E-03 |
| SLIT2 | slit homolog 2 | 9353 | 2.13E-07 | 6.30E-03 |
| PPP1R15A | protein phosphatase 1, regulatory subunit 15A | 23645 | 2.30E-07 | 6.80E-03 |
| SRPX2 | sushi-repeat-containing protein, X-linked 2 | 27286 | 2.31E-07 | 6.84E-03 |
| MMP3 | matrix metallopeptidase 3 | 4314 | 2.45E-07 | 7.23E-03 |
| AXUD1 | AXIN1 up-regulated 1 | 64651 | 2.66E-07 | 7.84E-03 |
| IL1B | interleukin 1, beta | 3553 | 3.01E-07 | 8.87E-03 |
| PRSS23 | protease, serine, 23 | 11098 | 3.23E-07 | 9.50E-03 |
| MARCH3 | PREDICTED: membrane-associated ring finger (C3HC4) 3 | 115123 | 3.25E-07 | 9.56E-03 |
| BCL11B | B-cell CLL/lymphoma 11B (zinc finger protein) | 64919 | 3.41E-07 | 1.00E-02 |
| POPDC2 | popeye domain containing 2 | 64091 | 3.47E-07 | 1.02E-02 |
| SPHK1 | sphingosine kinase 1 | 8877 | 3.49E-07 | 1.03E-02 |
| STOM | stomatin | 2040 | 3.49E-07 | 1.03E-02 |
| LOC645313 | PREDICTED: misc_RNA | 645313 | 3.64E-07 | 1.07E-02 |
| MAP1S | microtubule-associated protein 1S | 55201 | 3.76E-07 | 1.10E-02 |
| BEX1 | brain expressed, X-linked 1 | 55859 | 3.84E-07 | 1.13E-02 |
| LEPREL1 | leprecan-like 1 | 55214 | 4.04E-07 | 1.18E-02 |
| FLNC | filamin C, gamma | 2318 | 4.15E-07 | 1.22E-02 |
| LOC100133609 | PREDICTED: similar to membrane-associated ring finger 3 | 100133609 | 4.19E-07 | 1.23E-02 |
| FAM65C | family with sequence similarity 65, member C | 140876 | 4.20E-07 | 1.23E-02 |

| | | | | |
|---|---|---|---|---|
| FAT1 | FAT tumor suppressor homolog 1 | 2195 | 4.59E-07 | 1.34E-02 |
| ACTG2 | actin, gamma 2, smooth muscle, enteric | 72 | 4.60E-07 | 1.35E-02 |
| SIK1 | salt-inducible kinase 1 | 150094 | 4.70E-07 | 1.38E-02 |
| INSIG1 | insulin induced gene 1 | 3638 | 4.71E-07 | 1.38E-02 |
| RAB3B | RAB3B, member RAS oncogene family | 5865 | 4.81E-07 | 1.41E-02 |
| EFEMP1 | EGF-containing fibulin-like extracellular matrix protein 1 | 2202 | 4.97E-07 | 1.45E-02 |
| ODC1 | ornithine decarboxylase 1 | 4953 | 5.04E-07 | 1.47E-02 |
| TRIB1 | tribbles homolog 1 | 10221 | 5.18E-07 | 1.52E-02 |
| MAP2K3 | mitogen-activated protein kinase kinase 3 | 5606 | 5.50E-07 | 1.61E-02 |
| HMGB2 | high-mobility group box 2 | 3148 | 5.55E-07 | 1.62E-02 |
| QPCT | glutaminyl-peptide cyclotransferase | 25797 | 5.69E-07 | 1.66E-02 |
| ARC | activity-regulated cytoskeleton-associated protein | 23237 | 5.69E-07 | 1.66E-02 |
| TRAM2 | translocation associated membrane protein 2 | 9697 | 5.90E-07 | 1.72E-02 |
| SCD | stearoyl-CoA desaturase | 6319 | 6.09E-07 | 1.78E-02 |
| CITED2 | Cbp/p300-interacting transactivator 2 | 10370 | 6.46E-07 | 1.88E-02 |
| NSMCE4A | non-SMC element 4 homolog A | 54780 | 6.82E-07 | 1.98E-02 |
| HEBP1 | heme binding protein 1 | 50865 | 6.87E-07 | 2.00E-02 |
| C8ORF4 | chromosome 8 open reading frame 4 | 56892 | 7.32E-07 | 2.13E-02 |
| GJB2 | gap junction protein, beta 2, 26kDa | 2706 | 7.33E-07 | 2.13E-02 |
| B3GALNT1 | beta-1,3-N-acetylgalactosaminyltransferase 1 | 8706 | 7.44E-07 | 2.16E-02 |
| C9ORF169 | chromosome 9 open reading frame 169 | 375791 | 7.70E-07 | 2.24E-02 |
| GPR84 | G protein-coupled receptor 84 | 53831 | 7.83E-07 | 2.27E-02 |
| SNORD3D | small nucleolar RNA, C/D box 3D | 780854 | 8.14E-07 | 2.36E-02 |
| PPARG | peroxisome proliferator-activated receptor gamma | 5468 | 9.52E-07 | 2.76E-02 |
| EDN1 | endothelin 1 | 1906 | 9.63E-07 | 2.79E-02 |
| RNU6-1 | RNA, U6 small nuclear 1 | 26827 | 9.82E-07 | 2.84E-02 |
| ZC3H12A | zinc finger CCCH-type containing 12A | 80149 | 1.03E-06 | 2.96E-02 |
| LOC645166 | PREDICTED: similar to lymphocyte-specific protein 1 | 645166 | 1.03E-06 | 2.97E-02 |
| GPD1L | glycerol-3-phosphate dehydrogenase 1-like | 23171 | 1.04E-06 | 2.99E-02 |
| TOB1 | transducer of ERBB2, 1 | 10140 | 1.37E-06 | 3.93E-02 |
| MT1G | metallothionein 1G | 4495 | 1.53E-06 | 4.40E-02 |
| TOP2A | topoisomerase (DNA) II alpha 170kDa | 7153 | 1.57E-06 | 4.49E-02 |
| NFIL3 | nuclear factor, interleukin 3 regulated | 4783 | 1.57E-06 | 4.49E-02 |
| SLC6A15 | solute carrier family 6, member 15 | 55117 | 1.65E-06 | 4.72E-02 |
| ABCC2 | ATP-binding cassette, sub-family C (CFTR/MRP) | 1244 | 1.67E-06 | 4.77E-02 |
| GSTA4 | glutathione S-transferase A4 | 2941 | 1.68E-06 | 4.81E-02 |
| TIPARP | TCDD-inducible poly(ADP-ribose) polymerase | 25976 | 1.69E-06 | 4.83E-02 |
| ZSWIM4 | zinc finger, SWIM-type containing 4 | 65249 | 1.69E-06 | 4.83E-02 |
| MFAP4 | microfibrillar-associated protein 4 | 4239 | 1.71E-06 | 4.89E-02 |
| SHMT2 | serine hydroxymethyltransferase 2 | 6472 | 1.76E-06 | 5.04E-02 |
| NDRG1 | N-myc downstream regulated gene 1 | 10397 | 1.88E-06 | 5.35E-02 |
| MGC4677 | PREDICTED: hypothetical protein | 112597 | 1.88E-06 | 5.35E-02 |
| ACPP | acid phosphatase, prostate | 55 | 2.04E-06 | 5.81E-02 |
| H2AFJ | H2A histone family, member J | 55766 | 2.06E-06 | 5.87E-02 |
| TGM2 | transglutaminase 2 | 7052 | 2.10E-06 | 5.97E-02 |
| PSIP1 | PC4 and SFRS1 interacting protein 1 | 11168 | 2.12E-06 | 6.02E-02 |
| D2HGDH | D-2-hydroxyglutarate dehydrogenase | 728294 | 2.52E-06 | 7.13E-02 |
| COG5 | component of oligomeric golgi complex 5 | 10466 | 2.64E-06 | 7.47E-02 |
| S1PR3 | sphingosine-1-phosphate receptor 3 | 1903 | 2.67E-06 | 7.56E-02 |
| TREM1 | triggering receptor expressed on myeloid cells 1 | 54210 | 2.71E-06 | 7.66E-02 |
| SLC1A3 | solute carrier family 1 | 6507 | 2.94E-06 | 8.32E-02 |
| LOC388796 | hypothetical LOC388796 | 388796 | 2.95E-06 | 8.32E-02 |
| NMB | neuromedin B | 4828 | 3.26E-06 | 9.17E-02 |
| LOC400027 | PREDICTED: hypothetical gene supported by BC047417 | 400027 | 3.37E-06 | 9.49E-02 |
| DFNA5 | deafness, autosomal dominant 5 | 1687 | 3.47E-06 | 9.77E-02 |
| IGFBP1 | insulin-like growth factor binding protein 1 | 3484 | 3.49E-06 | 9.82E-02 |
