# Supplementary material for: Host mediated inflammatory influence on glioblastoma multiforme recurrence following high-dose ionizing radiation
Source: PLoS One. 2017 May 22;12(5):e0178155. doi: 10.1371/journal.pone.0178155 (PMC5439715; doi:10.1371/journal.pone.0178155)
Supplement: S2 Table — Statistically significant (FWER < 5%) gene sets up- or down-regulated compared to 0Gy. (PDF) [file pone.0178155.s002.pdf]

S2 Table. GSEA gene ontology analysis. Statistically significant (FWER < 5%) gene sets up- or down-regulated compared to 0Gy.

Comparison	Up or down-regulated	# of Gene Sets	Gene Set Name
8Gy day1	Up	5	actin polymerization and or depolymerization, epidermal growth factor receptoring pathway, extracellular region, extracellular region part, protein polymerization
16Gy day1	Up	3	cell matrix adhesion, cell substrate adhesion, extracellular region
8Gy day4	Up	29	anti apoptosis, behavior, chemokine activity, chemokine receptor binding, cytokine activity, defense response, extracellular region, extracellular region part, extracellular space, female pregnancy, G protein coupled receptor binding, humoral immune response, immune response, immune system process, inflammatory response, locomotory behavior, negative regulation of apoptosis, negative regulation of cell proliferation, negative regulation of developmental process, negative regulation of programmed cell death, positive regulation of cell proliferation, receptor binding, regulation of cell proliferation, response to chemical stimulus, response to external stimulus, response to stress, response to wounding, viral reproduction, viral reproductive process
16Gy day4	Up	27	anti apoptosis, behavior, chemokine activity, chemokine receptor binding, cytokine activity, defense response, extracellular region, extracellular region part, extracellular space, female pregnancy, G protein coupled receptor binding, growth factor activity, hematopoietin interferon classd200 domain cytokine receptor binding, humoral immune response, inflammatory response, locomotory behavior, negative regulation of apoptosis, negative regulation of cell proliferation, negative regulation of developmental process, negative regulation of programmed cell death, positive regulation of cell proliferation, receptor binding, regulation of cell proliferation, response to external stimulus, response to stress, response to wounding, viral reproductive process
8Gy day6	Up	15	behavior, chemokine activity, chemokine receptor binding, cytokine activity, extracellular region, extracellular region part, extracellular space, G protein coupled receptor binding, inflammatory response, locomotory behavior, positive regulation of cell proliferation, receptor binding, response to external stimulus, response to wounding, serine type endopeptidase inhibitor activity
16Gy day6	Up	19	behavior, chemokine activity, chemokine receptor binding, cytokine activity, defense response, extracellular region, extracellular region part, extracellular space, female pregnancy, G protein coupled receptor binding, inflammatory response, locomotory behavior, multi organism process, negative regulation of apoptosis, negative regulation of developmental process, positive regulation of cell proliferation, receptor binding, response to external stimulus, response to wounding
8Gy day35	Up	0	- - -
16Gy day35	Up	1	myosin complex
8Gy day1	Down	38	cell cycle checkpoint (GO:0000075), cell cycle (GO:0007049), cell cycle phase, cell cycle process, chromatin, chromatin binding, chromosomal part, chromosome, chromosome organization and biogenesis, chromosome segregation, chromosomepericentric region, condensed chromosome, condensed nuclear chromosome, DNA dependent DNA replication, DNA metabolic process, DNA packaging, DNA recombination, DNA repair, DNA replication, kinetochore, M phase, M phase of mitotic cell cycle, microtubule cytoskeleton organization and biogenesis, mitosis, mitotic cell cycle, nuclear chromosome, nuclear chromosome part, replication fork, response to DNA damage stimulus, ribonucleoprotein complex, ribonucleoprotein complex biogenesis and assembly, ribosome biogenesis and assembly, RNA polymerase activity, RNA processing, rRNA metabolic process, rRNA processing, spindle, spindle pole
16Gy day1	Down	51	cell cycle checkpoint (GO:0000075), cell cycle (GO:0007049), cell cycle phase, cell cycle process, chromatin, chromatin assembly or disassembly, chromatin binding, chromosomal part, chromosome, chromosome organization and biogenesis, chromosome segregation, chromosomepericentric region, condensed chromosome, DNA dependent DNA replication, DNA metabolic process, DNA packaging, DNA repair, DNA replication, double strand break repair, establishment and or maintenance of chromatin architecture, interphase, interphase of mitotic cell cycle, kinetochore, M phase, M phase of mitotic cell cycle, microtubule cytoskeleton organization and biogenesis, mitosis, mitotic cell cycle, mitotic sister chromatid segregation, mRNA metabolic process, nuclear chromosome, nuclear chromosome part, nuclear envelope, nuclear lumen, nuclear membrane, nuclear membrane part, nuclear pore, nucleoplasm, nucleotidyltransferase activity, pore complex, response to DNA damage stimulus, ribonucleoprotein complex biogenesis and assembly, ribosome biogenesis and assembly, RNA binding, RNA polymerase activity, RNA processing, rRNA metabolic process, rRNA processing, sister chromatid segregation, spindle, spindle pole

8Gy day4	Down	11	amino acid transport, carboxylic acid metabolic process, cofactor metabolic process, mitochondrion, organic acid metabolic process, oxidoreductase activity, oxidoreductase activity acting on CH-OH group of donors, oxidoreductase activity (GO:0016616), ribosomal subunit, ribosome, structural constituent of ribosome
16Gy day4	Down	19	carboxylic acid metabolic process, coenzyme metabolic process, cofactor metabolic process, mitochondrial inner membrane, mitochondrial membrane part, mitochondrial part, mitochondrion, organelle inner membrane, organic acid metabolic process, oxidoreductase activity, oxidoreductase activity acting on CH-OH group of donors, oxidoreductase activity acting on the aldehyde or oxo group of donors, oxidoreductase activity acting on the ch ch group of donors, oxidoreductase activity (GO:0016616), ribosomal subunit, ribosome, RNA binding, translation factor activity nucleic acid binding, translation initiation factor activity
8Gy day6	Down	9	amine transmembrane transporter activity, amino acid transmembrane transporter activity, amino acid transport, electron transport (GO:0006118), glutathione transferase activity, oxidoreductase activity, oxidoreductase activity acting on CH-OH group of donors, oxidoreductase activity (GO:0016616), translation initiation factor activity
16Gy day6	Down	6	amine transmembrane transporter activity, amine transport, amino acid transmembrane transporter activity, establishment of organelle localization, oxidoreductase activity acting on CH-OH group of donors, oxidoreductase activity (GO:0016616)
8Gy day35	Down	0	- - -
16Gy day35	Down	9	chromosome pericentric region, coenzyme metabolic process, kinetochore, RNA binding, mitotic cell cycle, structural constituent of ribosome, nuclear envelope, nuclear membrane part, M phase of mitotic cell cycle
