# Supplementary material for: Host mediated inflammatory influence on glioblastoma multiforme recurrence following high-dose ionizing radiation
Source: PLoS One. 2017 May 22;12(5):e0178155. doi: 10.1371/journal.pone.0178155 (PMC5439715; doi:10.1371/journal.pone.0178155)
Supplement: S3 Table — The top three enrichment groups found from the 273 identifiable genes with statistical significance. (PDF) [file pone.0178155.s003.pdf]

S3 Table. Functional gene classification using DAVID. The top three enrichment groups found from the 273 identifiable genes with statistical significance.

Group	Enrichment Score*	Kappa**	Gene name
IL-1 Family	8.851	**0.97**	**IL1F10: interleukin 1 family, member 10 (theta)**
		**0.94**	**IL1F9: interleukin 1 family, member 9**
		**0.89**	**IL1F5: interleukin 1 family, member 5 (delta)**
		**0.71**	**IL1F8: interleukin 1 family, member 8 (eta)**
		0.57	IL1RN; interleukin 1 receptor antagonist
		0.46	IL33: interleukin 33
Chemokine Family (C-C and C-X-C motif ligand)	7.078	**0.83**	**CXCL2: chemokine (C-X-C motif) ligand 2**
		**0.82**	**CCL20: chemokine (C-C motif) ligand 20**
		**0.81**	**CCL3L3/L1: chemokine (C-C motif) ligand 3-like 3/1**
		**0.80**	**CXCL5: chemokine (C-X-C motif) ligand 5**
		**0.77**	**CXCL1: chemokine (C-X-C motif) ligand 1**
		0.59	CCL3: chemokine (C-C motif) ligand 3
		0.45	IL8: interleukin 8
		0.41	GDF15: growth differentiation factor 15
		0.38	CCL5: chemokine (C-C motif) ligand 5
		0.35	IL1RN: interleukin 1 receptor antagonist
Histone Family	3.172	**0.93**	**HIST1H2BD: histone cluster 1, H2bd**
		**0.92**	**HIST2H2AA3/4: histone cluster 2, H2aa3; H2aa4**
		**0.91**	**H2AFJ: H2A histone family, member J**
		**0.90**	**HIST1H2AC: histone cluster 1, H2ac**
		**0.90**	**HIST2H2AC: histone cluster 2, H2ac**
		**0.88**	**HIST1H2BF: histone cluster 1, H2bi**
		**0.88**	**HIST2H2BE: histone cluster 2, H2be**
		0.73	HIST1H4J: histone cluster 1, H4l
		0.73	H1F0: H1 histone family, member 0
		0.51	HJURP: Holliday junction recognition protein

*Geometric mean of the overall group significance based on gene EASE scores (modified Fisher Exact P-Value) in -log scale.

Gene-to-gene Similarity Score: **Very high (0.75-1), High (0.5-0.75), Moderate (0.25-0.5), Low (<0.25)
